# Role of Si/dielectric interface traps on the reliability of tree-shaped complementary FETs for sub-3 nm technology nodes

**DOI:** 10.1038/s41598-026-57907-2

**Published:** 2026-06-16

**Authors:** S. Shreyas Thanthri, Sresta Valasa, Venkata Ramakrishna Kotha, B. Mounika, Narendar Vadthiya

**Affiliations:** 1 Manipal Institute of Technology Bengaluru, Manipal Academy of Higher Education, Manipal, India; 2 Department of ECE, National Institute of Technology Warangal, Warangal, 506004 India; 3 Department of ECE, SR University, Warangal, Telangana India

**Keywords:** Si/dielectric interface, Interface density, Sub- 3nm, CFETs, Reliability, CMOS inverter, Energy science and technology, Engineering, Materials science, Nanoscience and technology, Physics

## Abstract

Complementary (C) FETs enable superior scaling and electrostatics for sub-3 nm nodes but the increased Si/dielectric interface reliability a critical concern, and its impact on Tree-shaped CFETs is investigated for the first time in this work. The Si/dielectric interface trap density (N_it_) is varied from 1 to 9 × 10^12^ cm^− 2^ for both acceptor and donor traps to evaluate their influence on digital, analog, RF, and circuit characteristics. With increasing N_it_, in the n-type (p-type) device, acceptor (donor) traps repel electrons (holes), reducing I_ON_ while suppressing I_OFF_ by ~ 5 orders, improving the I_ON_/I_OFF_ ratio. In p-type (n-type) device acceptor (donor) traps attract holes (electrons), increasing I_ON_ by ~ 68% (~ 58%) but reducing the ratio to 10^2^ due to higher leakages. Notably, when the N_it_ exceeds 4 × 10^12^ cm^− 2^, the switching ratio deteriorates below 10^4^ for donor (acceptor) traps in the n-type (p-type) device due to pronounced trap-assisted leakage. The findings demonstrate that the Tree-shaped CFET maintains strong electrostatic integrity till ≤ 4 × 10^12^ cm^− 2^ and stable analog/RF behavior even under severe Si/dielectric interface trap perturbations. The Tree-CFET CMOS inverter shows robust circuit behavior with ~ 2.9 ps delay at moderate trap densities, highlighting the need for precise interface engineering for sub-3 nm nodes.

**Supplementary Information:**

The online version contains supplementary material available at 10.1038/s41598-026-57907-2.

## Introduction

As CMOS technology continues to scale toward sub-3 nm technology nodes, conventional lateral device architectures such as Forksheet (FS) FETs face increasing limitations in achieving higher logic density^1^. Hence, further scaling requires vertically stacked transistor concepts such as Complementary (C) FETs to sustain density scaling and device integration^2,3^ which vertically stack nMOS and pMOS transistors within the same footprint, enabling reduced standard cell height and higher transistor density. The vertical integration also shortens inter-device routing distances, thereby reducing parasitic capacitances and improving switching performance^4^. However, most CFET implementations employ conventional NS channels, whose planar geometry limits further enhancement of effective channel perimeter and current drive within a compact footprint. Beyond standard logic switching, advanced variants of nanoscale transistors are increasingly being leveraged for multi-disciplinary paradigms, including high-frequency signal amplification, photonic integration, and ultra-sensitive biomolecular or gas detection^5–7^. Due to their superior electrostatic control and highly tunable electrical properties, these modern architectures serve as vital building blocks for low-power processing units, hardware-level security primitives, and highly integrated multi-functional systems^8,9^. In this view, advanced channel geometries such as the Tree-shaped channel have been explored^10–12^. This structure integrates stacked nanosheets with fin-like inter-bridges connecting adjacent NS, forming a conduction network that increases the effective channel perimeter and improves current distribution^13,14^. When incorporated into a CFET architecture, the Tree-shaped channel enhances drive current and electrostatic control without increasing lateral dimensions, making it highly suitable for vertically integrated devices. Consequently, the Tree-shaped CFET architecture combines the density benefits of CFET with the performance advantages of advanced channel engineering.

On the other hand, as device dimensions approach the sub-3 nm regime, reliability and variability issues become increasingly critical. Beyond basic electrostatic control, long-term device reliability and structural immunity to aging and hot-carrier degradation remain key challenges in advanced nanoscale architectures. Furthermore, geometric engineering, such as channel length modulation, can mitigate severe hot-carrier injections by altering internal peak electric fields, ensuring better reliability under varying temperature profiles^15,16^. Incorporating advanced configurations like material-driven junctions, macaroni-channel designs, or hetero-interfaced geometries can further enhance the structural robustness against thermal and hot-carrier-induced aging^17,18^. The presence of interface trap variations along the Si/dielectric boundary can significantly alter electrical metrics playing a dominant role^19–23^. Owing to the high surface-to-volume ratio in GAA and CFET structures, these traps significantly influence electrostatics and carrier transport. Interface states, originating from dangling bonds and process-induced defects, are generally classified as acceptor and donor traps, which introduce localized energy levels in the bandgap and modify threshold voltage, drive current, and channel electrostatics^24–26^. Although prior studies have investigated interface trap effects in nanosheet FETs, FSFETs, TreeFETs, and Negative Capacitance (NC) FETs, the present work differs significantly in terms of device architecture, electrostatic configuration, and reliability focus. A detailed benchmarking table with prior literature is included below in Table 1 to differentiate the study. To the best of the authors’ knowledge, this is the first comprehensive investigation of Si/dielectric interface trap effects in a vertically stacked Tree-shaped CFET architecture considering both acceptor and donor traps across digital, analog, RF, and circuit domains simultaneously. Unlike conventional NS and FS devices, the proposed Tree-shaped CFET employs a vertically integrated complementary architecture with enhanced gate-controlled interface area and tree-shaped channel topology, resulting in fundamentally different trap-carrier electrostatic interactions. Furthermore, previous interface trap studies have primarily focused on either digital characteristics or isolated analog metrics, whereas the present work provides a unified reliability assessment including digital, analog, RF parameters, and CMOS inverter-level performance under varying trap polarity and density conditions. Therefore, this work proposes a novel Tree-shaped CFET architecture at the sub-3 nm node and presents the first comprehensive investigation of Si/dielectric interface trap effects considering both acceptor and donor traps, providing key insights into its digital, analog, RF, and circuit suitability for next-generation high-density CMOS technologies.

*The novel contributions of the work as follows*: (i) A comprehensive analysis of acceptor and donor interface traps on digital performance is performed, revealing strong carrier–trap electrostatic interactions and identifying a critical Si/dielectric trap density (~ 4 × 10^12^ cm^− 2^) beyond which switching performance significantly degrades. (ii) The analog and RF characteristics are evaluated, demonstrating limited degradation in g_m_, A_V_, and C_GG_, indicating strong electrostatic robustness of the Tree-shaped CFET structure under heavy Si/dielectric interface trap perturbations. (iii) Circuit-level validation using a CFET-based CMOS inverter confirms stable noise margins, minimal propagation delay (~ 2.9 ps), and reliable switching behavior in the presence of Si/dielectric interface traps.

**Table 1:** Benchmarking of the proposed device with previous literature.

Ref.	Device	Trap-type	Nit range	I_ON_	I_OFF_	I_ON_/I_OFF_	SS	Limitations
^24^	NC p-FinFET	Donor	1–4 × 10^12^ cm^−2^	12 uA	~ 10^–10^ A	~ 10^4^	–	No analog/RF performance is reported
^27^	NWFET	Acceptor	1 × 10^12^ cm^−2^	~ 130 uA	~ 10^–13^ A	~ 10^9^	68.85	Donor traps influence is not reported
^27^	NSFET	Acceptor	1 × 10^12^ cm^−2^	~ 120 uA	~ 10^–14^ A	~ 10^9^	65.23	Donor traps influence is not reported
^28^	JL-NSFET	Acceptor	~ 10^12^ cm^−2^ - ~10^14^ cm^−2^	~ 35 uA	–	–	–	Only trap sensitivity is reported.
^28^	JL-NSFET	Donor	~ 10^12^ cm^−2^ - ~10^14^ cm^−2^	60 uA	–	–	–	Only trap sensitivity is reported.
^29^	FSFET	Acceptor/ Donor	1 × 10^12^ cm^−2^	~ 10^− 4^	n-~10^–10^ – 10^–11^ p-~10^–10^ – 10^− 9^	~ 10^6^ - ~10^7^	–	No analog/RF, and circuit analysis reported
^30^	NCFET	Donor	1 × 10^12^ cm^−2^	2.68 mA/um	–	–	–	Circuit analysis is not reported
^30^	NCFET	Acceptor		1.40 mA/um	–	–	–	Circuit analysis is not reported
This work	Tree-CFET	Acceptor/ Donor	1–9 × 10^12^ cm^−2^	varies	varies	varies	varies	–

## Device setup and simulation details

**Fig. 1:**
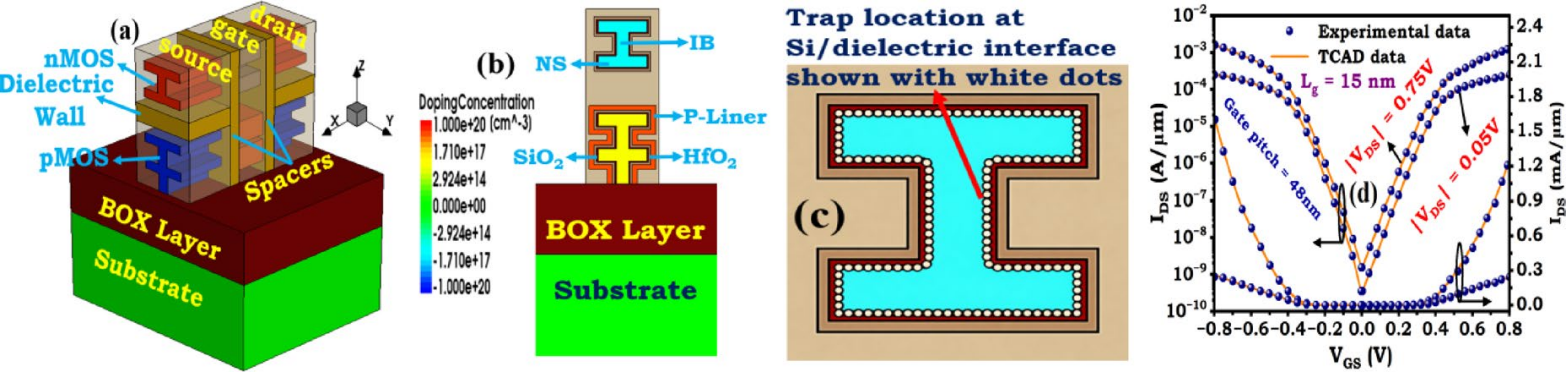
Proposed Tree-shaped CFET (**a**) 3D view (**b**) 2D Tree channel with doping profile (**c**) Interface traps at Si/dielectric interface shown for upper Tree channel. The similar traps also exist for the lower Tree channel. (**d**) Calibration with experimental data^37^.

The proposed Tree-shaped CFET designed is simulated using the Synopsys Sentaurus TCAD^31^, enabling rigorous three-dimensional electrostatic and carrier transport analysis. Both 3D and 2D cross-sectional views of the architecture are presented in Fig. 1a,b respectively to clearly illustrate the vertical stacking configuration, Tree-shaped channel topology, and gate stack arrangement. Additionally, detailed doping profiles for the source/drain and channel regions are provided, along with explicit identification of the trap locations at the Si/dielectric interfaces in Fig. 1c. The structural parameters of the proposed CFET, designed according to the IRDS 3 nm node guidelines^32^, are summarized in Table 2. The gate length (L_g_) is set to 15 nm with a contacted gate pitch (CGP) of 48 nm to ensure compatibility with advanced standard cell layouts. The nanosheet thickness (T_NS_) and width (W_NS_) are chosen as 5 nm and 20 nm, respectively. To realize the tree topology, inter-bridge (IB) channels are introduced with width and heights of (W_IB_ = 5 nm and H_IB_ = 10 nm), providing additional conduction paths and increased channel periphery. A 5 nm spacer is used on both source and drain sides to reduce parasitic capacitance. The vertical spacing between stacked nMOS and pMOS devices is maintained at 20 nm, while a 10 nm dielectric wall is incorporated to ensure electrical isolation and suppress inter-device electrostatic coupling in the Tree-shaped CFET.

**Fig. 2:**
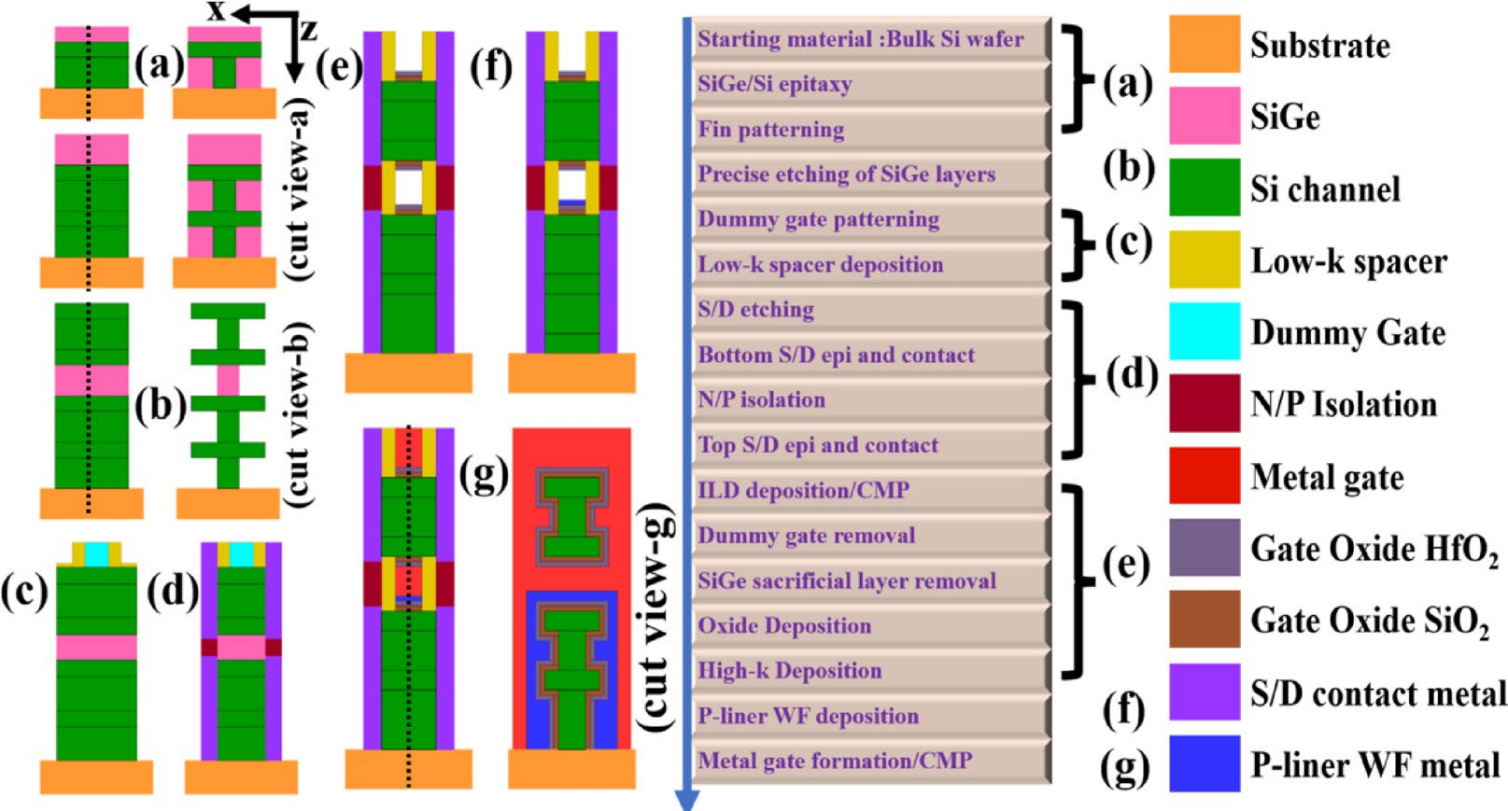
Fabrication process flow of proposed CFET device. The dashed line in black represents the cut views of (**a**), (**b**), (**g**) steps.

A high-k/metal gate stack is employed to achieve aggressive EOT scaling. A 0.55 nm SiO_2_ dielectric interfacial layer preserves interface quality, followed by 1.12 nm HfO_2_ to reduce gate leakage while maintaining strong electrostatic control^33^, resulting in an EOT of ~ 0.75 nm. A 2 nm p-liner assists work-function tuning for the pMOS device^34^, with metal work functions of 4.6 eV (nMOS) and 4.64 eV (pMOS) for balanced threshold voltages. The source/drain regions are heavily doped (1 × 10^20^ cm^− 3^) to reduce series resistance, while the channel is lightly doped (1 × 10^16^ cm^− 3^) to preserve mobility. To analyze reliability, the Si/dielectric interface trap density (N_it_) is varied from 1 × 10^11^/cm^2^ to 9 × 10^12^/cm^2^^35,36^, while fixed interface charges are excluded since they cannot accurately capture the electrostatic and subthreshold effects of dynamic interface traps. Furthermore, the CFET device is calibrated against reported experimental data^37^ as visualized in Fig. 1d to ensure realistic and reliable performance prediction. Key parameters, including velocity saturation coefficients, mobility degradation factors, work function values, and other transport-related coefficients, are carefully tuned within physically acceptable limits to achieve close agreement with experimentally demonstrated characteristics. The CMOS compatible process flow for the proposed Tree-shaped CFET is shown in Fig. 2.

**Table 2:** Specifications of Tree-CFET following IRDS-3-nm guidelines.

Device specifications	Values
Contact gate pitch (CGP)	48 nm
Gate length (L_g_)	15 nm
IB width (W_IB_)	5 nm
IB height (H_IB_)	10 nm
NS thickness (T_NS_)	5 nm
NS width (W_NS_)	20 nm
Spacer length	5 nm
Spacing between nMOS and pMOS	20 nm
SiO_2_ Thickness	0.55 nm
HfO_2_ Thickness	1.12 nm
P-liner Thickness	2 nm
Contact resistivity	7 Ω.µm^2^
Sheet silicide resistivity	7.5 Ω/sq.
Effective oxide thickness (EOT)	0.75 nm
Work Function n/p	4.6 eV/4.64 eV
S/D doping	1 × 10^20^/cm^3^
Channel doping	1 × 10^16^/cm^3^
Si/dielectric interface trap density (N_it_) variation	1 × 10^11^/cm^2^ to 9 × 10^12^/cm^2^

The proposed architecture is fundamentally consistent with the latest research related to vertically stacked nanosheet and CFETs, which are being developed to implement CMOS technology below 3 nm. The latest research results related to stacked nanosheet transistors, forksheet field effect transistors, and monolithic CFET technology have shown that the concept of multi-layer epitaxial growth, selective channel release, self-aligned gates formation, and vertical stacking of complementary technology using EUV lithography is feasible^38–41^. The major problem in fabrication is associated with the need for proper alignment of the stacked nanosheet channels and the interbridge areas. As this design concept uses interconnected tree-like channels, any misalignment at the nano level in lithographic or epitaxial processes will result in a change in gate wrapping and electrostatic field effects, resulting in variations in the threshold voltage and electrostatic parasitics. This kind of alignment problem was also observed in FSFET and CFET designs because of extreme close contact gate pitch and spacer dimension at the 3-nm node level. Nevertheless, modern self-aligned gate and inner spacer techniques used in nanosheet and FSFET devices have helped to address this problem^42–45^. The next integration problem in the context of process integration is associated with creating the interbridge regions connecting the adjacent nanosheets. It appears that there is a need for highly controlled and accurate sacrificial layer etching and channel opening processes similar to those used for the stacked GAA nanosheet FET devices. Here, maintaining the mechanical integrity of the suspended interbridge region while performing selective etch and deposition is crucial to prevent any distortion and electric field asymmetry. In addition, it is necessary to use conformal deposition techniques in relation to both high-k dielectric material and the metal gate to achieve a good degree of gate controllability without electric field crowding^46–49^.

To accurately capture carrier transport and electrostatics in the proposed Tree-shaped CFET, comprehensive physical models are implemented within the TCAD framework. Carrier transport is described using the drift-diffusion model, solving the coupled poisson and continuity equations to obtain reliable I-V characteristics^50^, while Fermi-Dirac statistics account for carrier distribution in highly doped and confined regions^51^^[,52^. Recombination is modeled using Shockley-Read-Hall (SRH) and Auger recombination to capture trap-assisted and high-injection effects. Mobility degradation is modeled using the Philips unified mobility model for doping dependence, the Lombardi model for surface scattering, and the IAL mobility model for inversion and accumulation layers, along with a thin-layer mobility model to represent transport in ultra-thin channels^53–55^. High-field velocity saturation is incorporated using the Caughey-Thomas model. Quantum confinement is captured using the Modified Local Density Approximation (MLDA)^56^, while Old Slotboom bandgap narrowing accounts for heavily doped regions^57^. Additionally, high-k gate stack effects are modeled through Remote Coulomb Scattering (RCS)^58^, Remote Phonon Scattering (RPS)^59^, and Remote Dipole Scattering (RDS) to accurately represent mobility degradation due to dielectric interactions^60^.

## Results and discussions

### Impact of digital performance at Si/dielectric interface

**Fig. 3:**
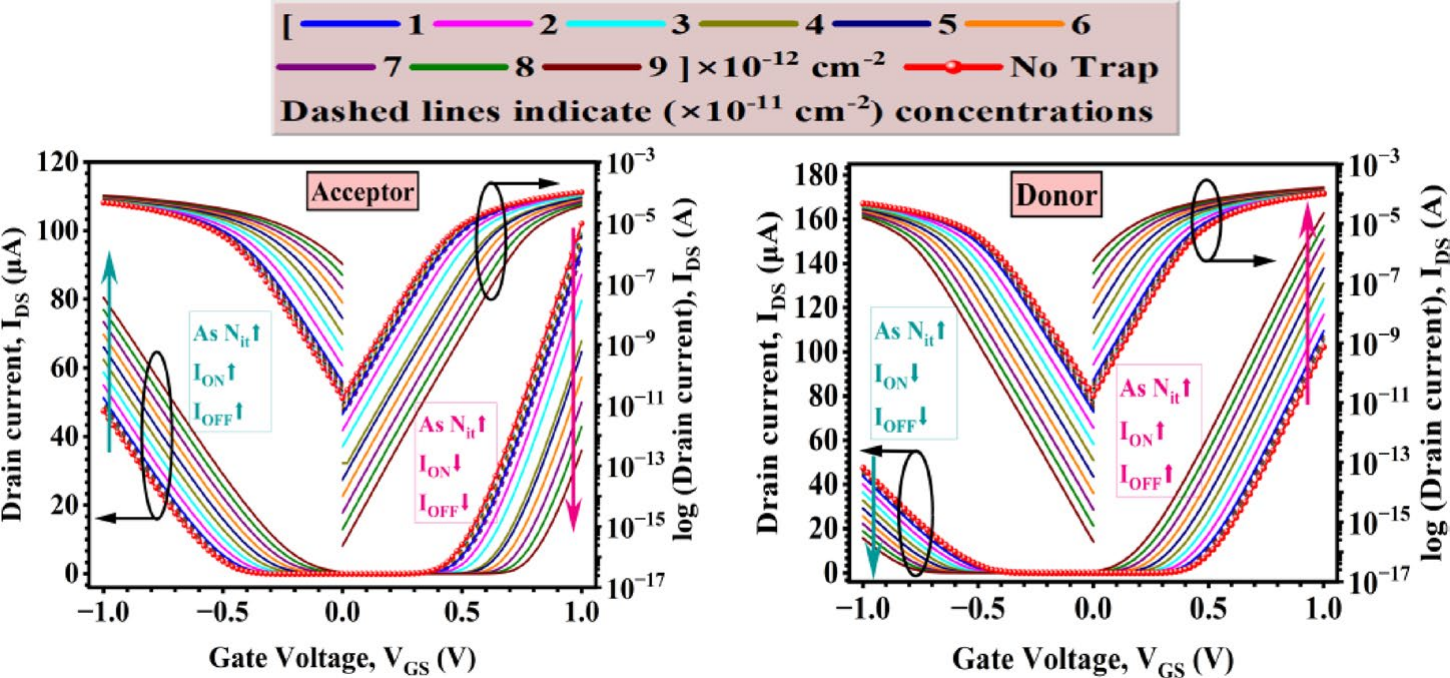
Influence of acceptor and donor N_it_ on I_D_-V_GS_.

**Fig. 4:**
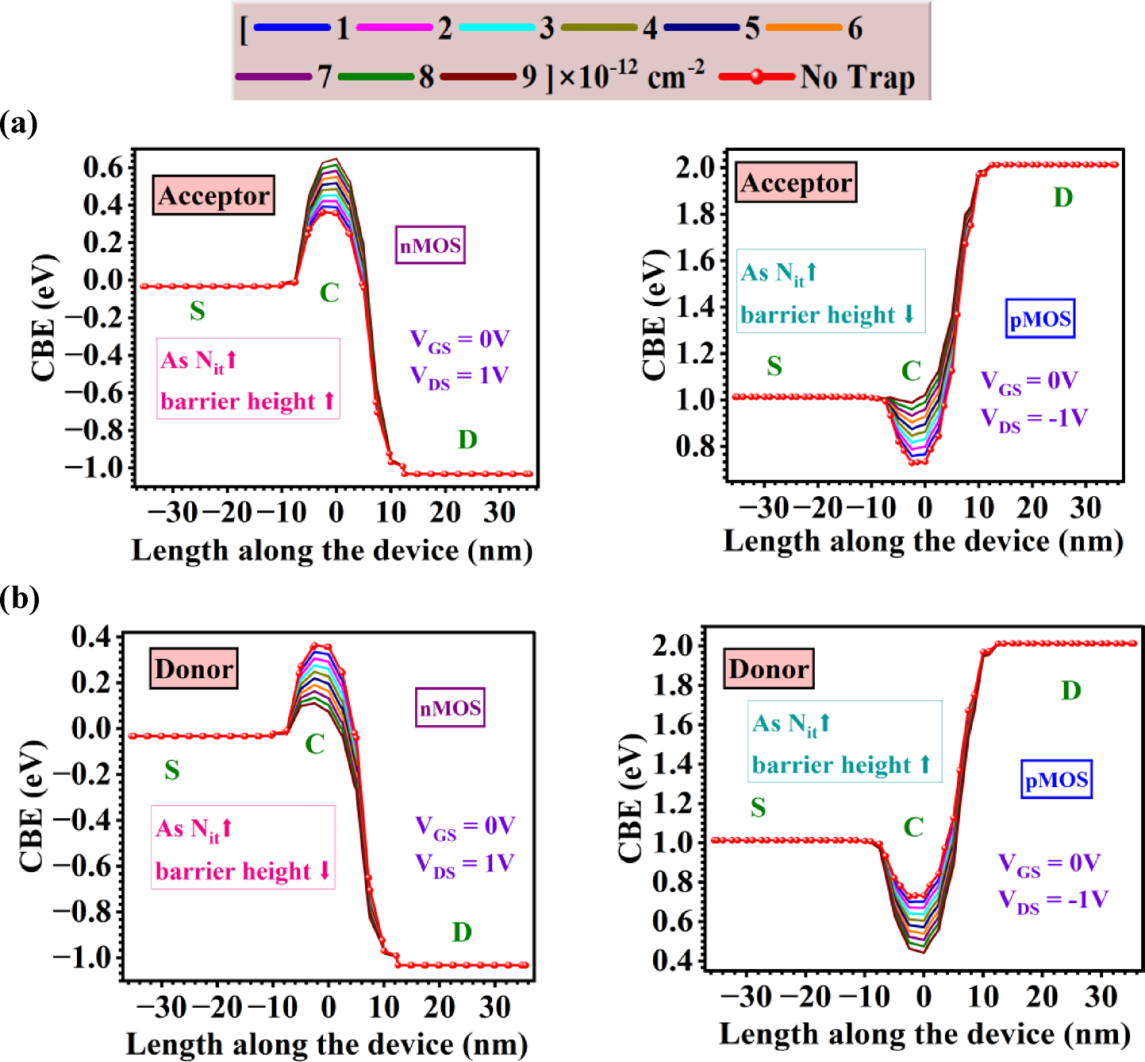
Conduction band energy (CBE) profile consisting of (**a**) acceptor and (**b**) donor traps.

**Fig. 5:**
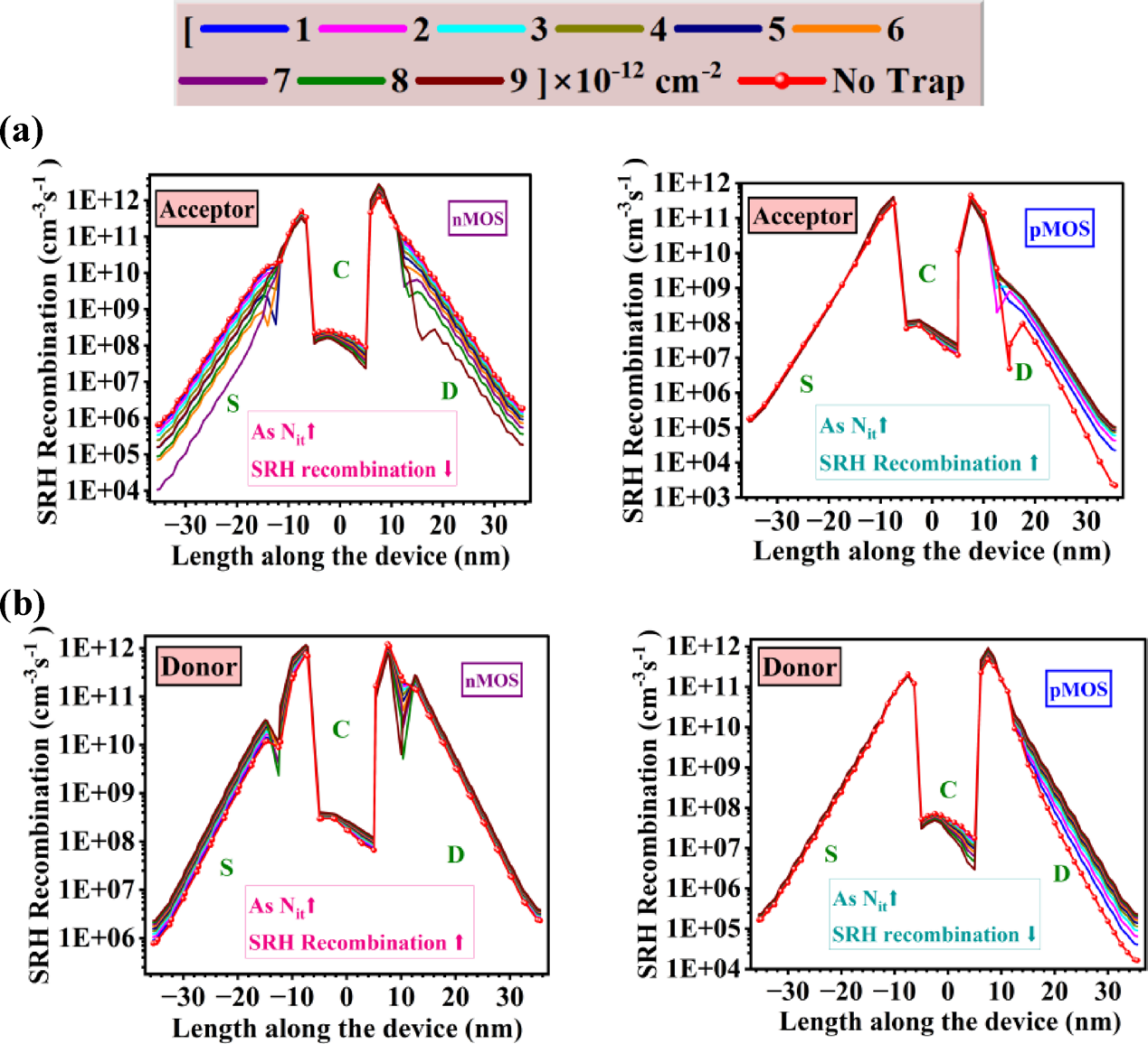
SRH Recombination rate profile consisting of (**a**) acceptor and (**b**) donor traps.

The influence of interface traps on the I-V characteristics of the proposed Tree-shaped CFET architecture is shown in Fig. 3. For the n-type device, under acceptor trap conditions, the traps capture channel electrons and become negatively charged. The accumulation of these negative charges near the Si/dielectric interface introduces a repulsive coulombic potential barrier for electrons, which weakens the gate-induced inversion layer. This results in a reduction in the effective electron concentration in the channel and also introduces coulomb scattering centers, degrading the electron current density and carrier mobility. Consequently, the I_ON_ decreases as the trap density increases. At relatively low trap densities from 1 × 10^− 11^ cm^− 2^ to 1 × 10^− 12^ cm^− 2^, the reduction in on current (I_on_) is modest from 101.3 µA to 94.6 µA (~ 6.61%) because the number of trapped charges is still small compared with the inversion charge induced by the gate. However, when the trap density increases further (≥ 1 × 10^− 12^ cm^− 2^), the accumulated negative charges significantly distort the channel potential, leading to strong mobility degradation and partial depletion of the inversion layer, which results in the substantial reduction in I_on_ observed from 94.6 µA to 35.9 µA (~ 62%). This can be also verified with the reduction in electron current density which is shown in supplementary information. Simultaneously, these negative charges strengthen the source-to-channel potential barrier in the off state by depleting residual electrons (as shown in Fig. 4a), and decreasing the recombination rate (Fig. 5a) thereby significantly suppressing the off current (I_OFF_). As the Si/dielectric trap density increases beyond 1 × 10^− 12^ cm^− 2^, the electrostatic perturbation produced by the trapped charge becomes comparable to the gate-controlled inversion charge, leading to a drastic suppression in I_OFF_ producing ~ 5-decade change. Also, the device without traps exhibits higher I_on_ and relatively larger I_OFF_ attributed to the channel electrostatics being unperturbed by trap-induced charges.

In contrast, the p-type device exhibits an opposite trend under acceptor trap conditions. Since acceptor traps capture electrons, the trapped negative charges effectively enhance hole accumulation in the channel through electrostatic attraction, thereby strengthening the inversion layer of holes. As a result, the channel conductivity improves, leading to an increase in I_on_ as the Si/dielectric trap concentration increases. At lower trap densities the enhancement is limited because the induced electrostatic assistance is small, resulting in only a slight increase from 47.8 µA to 51.2 µA (~ 7.1%). As the trap density becomes high (≥ 1 × 10^− 12^ cm^− 2^), the increased negative charge density significantly strengthens hole confinement near the interface, which increases the channel charge density and leads to a significant rise in I_on_ up to 80.4 µA (~ 57%). The increase in hole current density also confirms this behavior as indicated in supplementary information. However, the same electrostatic attraction lowers the source-channel energy barrier in the off state (Fig. 4a) and increases the recombination rate (Fig. 5a) which facilitates trap-assisted carrier generation and tunneling, and significantly increases the I_OFF_ till 4 decades. At higher Si/dielectric trap concentrations beyond 1 × 10^− 12^ cm^− 2^, the strong negative interface charge effectively induces partial hole accumulation even under subthreshold bias, resulting in a dramatic increase in I_OFF_. This explains why the device without traps exhibits the lowest leakage current compared with all acceptor trap cases for the p-type device. It is also noteworthy that even though the p-type I_on_ increases with acceptor trap density, the overall magnitude remains lower than that of the n-type device due to the intrinsically lower hole mobility in silicon compared with electron mobility.

A complementary behavior is observed when donor Si/dielectric interface traps are introduced. Donor traps capture holes and become positively charged, which strongly influences the electrostatics of the channel. In the n-type device, these positive charges attract electrons toward the interface and effectively reinforce the gate electric field responsible for electron inversion. This leads to an increase in electron carrier current density (supplementary information) within the channel, thereby strengthening the conductive path between source and drain. At lower trap densities, the improvement is modest (~ 6.4%) because the induced electrostatic enhancement is limited. However, at higher Si/dielectric interface trap densities (≥ 1 × 10^− 12^ cm^− 2^), the accumulated positive charges significantly intensify the electron confinement near the interface and reduce the effective channel resistance, resulting in a substantial increase in I_on_ from 102.8 µA to 162.7 µA (~ 58.2%). However, the same positive charges reduce the source-channel potential barrier in the off state as shown in Fig. 4b and increase the recombination rate (Fig. 5b) thereby promoting electron accumulation near the interface, leading to a substantial increase of ~ 4 decade variation in I_OFF_.

The effect becomes particularly pronounced at higher Si/dielectric interface trap densities where the accumulated positive interface charge strongly perturbs the channel potential and increases trap-assisted leakage pathways. Conversely, in the p-type device under donor trap conditions, the positive interface charge repels hole carriers away from the channel interface, which weakens the hole inversion layer and degrades the channel conductivity in the on state, resulting in a reduction in I_ON_. This reduction is modest at lower trap densities (~ 7.21%) but becomes significant by ~ 64.3% when the trap concentration exceeds (≥ 1 × 10^–12^ cm^-2^), where the Si/dielectric interface charge density becomes comparable to the inversion charge density and strongly perturbs the channel electrostatics. At the same time, the electrostatic repulsion of holes increases the source-channel potential barrier in the off state as shown in Fig. 4b and decreases the recombination rate (Fig. 5b) which suppresses the residual carrier concentration within the channel. This enhanced depletion significantly reduces the leakage current, driving I_OFF_ toward the femtoampere regime (~ 5 decade reduction) at higher Si/dielectric interface trap concentrations.

**Fig. 6:**
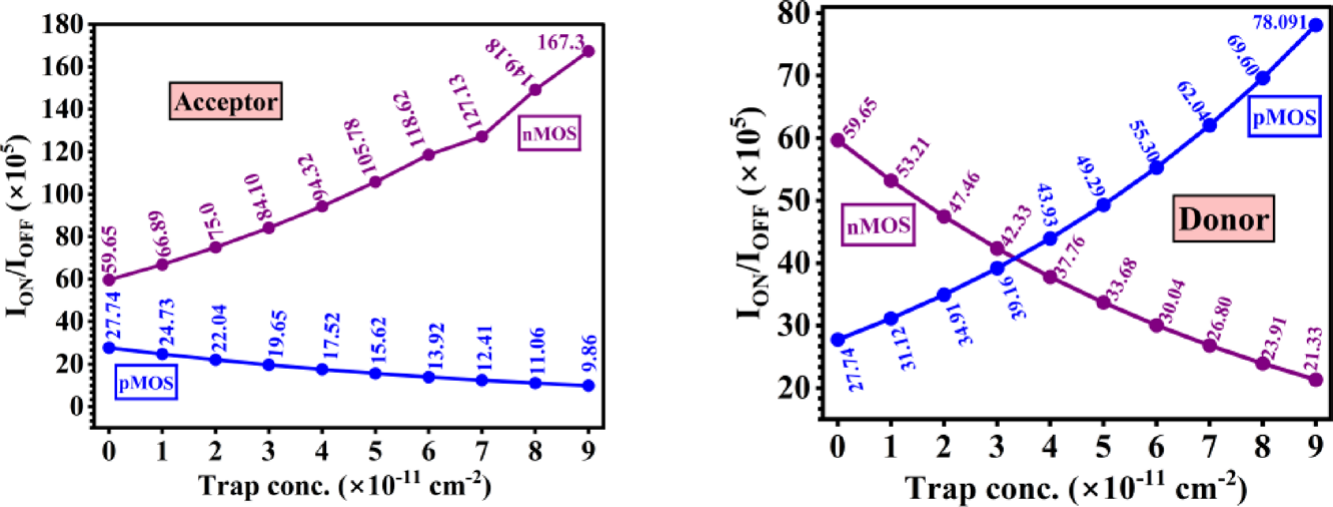
Influence of acceptor and donor N_it_ on I_ON_/I_OFF_ ratio (switching ratio). Here 0 indicates No Trap case. (1–9) ×10^− 12^ cm^− 2^ N_it_ is included in supplementary information as Tables S1 and S2.

The variation in the I_ON_/I_OFF_ ratio is plotted in Fig. 6. For the n-channel, acceptor traps (negative charge) repel electrons and strongly suppress leakage, resulting in a significant improvement in the switching ratio from 10^6^ to 10^11^, whereas donor traps (positive charge) attract electrons and increase leakage, degrading the ratio from 10^6^ to 10^2^. Conversely, for the p-channel, acceptor traps attract holes and increase leakage, reducing the ratio to 10^6^ to 10^2^, while donor traps repel holes and suppress leakage, improving the ratio up to 10^6^ to 10^10^. Thus, traps that repel the dominant channel carriers enhance the switching ratio, whereas traps that electrostatically attract carriers deteriorate it by increasing subthreshold leakage. It should be particularly noted that the I_ON_/I_OFF_ ratio degrades significantly (< 10^4^) when N_it_ exceeds approximately $\:4\times\:{10}^{12}{\hspace{0.17em}}{\mathrm{cm}}^{-2}$ (acceptor traps in nMOS and donor traps in pMOS) because the trap-induced electrostatic field becomes sufficiently strong to perturb the channel surface potential and subthreshold barrier profile. The I_OFF_ in ultra-scaled devices can be approximately expressed as^61^:

$\:{I}_{OFF}\propto\:exp\left(-\frac{q{\phi\:}_{B}}{kT}\right)$where $\:{{\upphi\:}}_{\mathrm{B}}$ is the effective source-to-channel barrier height. At higher interface trap densities, the trapped charges introduce an additional electrostatic potential ($\:{\Delta\:}{{\upphi\:}}_{\mathrm{t}\mathrm{r}\mathrm{a}\mathrm{p}}$) near the Si/dielectric interface, modifying the barrier as $\:{\phi\:}_{B,eff}={\phi\:}_{B}-\varDelta\:{\phi\:}_{trap}\:$for traps that electrostatically attract the dominant carriers. Consequently, even a small reduction in barrier height (Fig. 4) produces an exponential increase in subthreshold leakage current due to the exponential dependence of I_OFF_ on $\:{{\upphi\:}}_{\mathrm{B}}$. Furthermore, the large density of interface states enhances SRH trap-assisted generation and tunneling pathways (Fig. 5), which additionally contribute to leakage conduction. Although I_ON_ changes moderately with increasing N_it_, the exponential increase in I_OFF_ dominates at higher trap densities, causing the switching ratio to deteriorate below 10^4^.

**Fig. 7:**
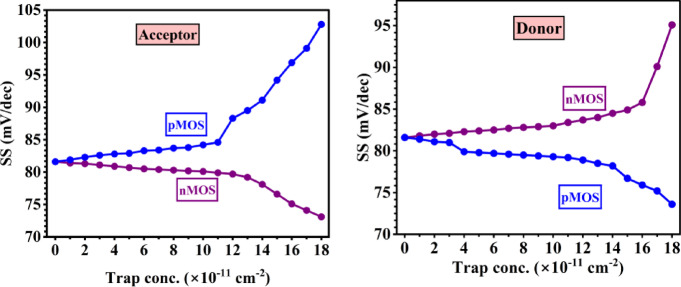
Influence of acceptor and donor N_it_ on subthreshold swing (SS). Here 0 indicates No Trap case.

The variation in subthreshold swing (SS) is visualized in Fig. 7. In the absence of traps, the device exhibits an SS of 81.6 mV/dec, which is governed by the intrinsic capacitive coupling between the gate oxide and the channel. The presence of Si/dielectric interface traps introduces additional charge states that modify the channel electrostatics and alter the effective capacitance in the subthreshold regime. For the n-channel, acceptor traps capture electrons and become negatively charged, repelling electrons and enhancing channel depletion, thereby strengthening the source-channel barrier and improving SS from 81.6 mV/dec to 73.1 mV/dec. In contrast, the same negative charges attract holes in the p-channel, weakening gate control and increasing trap-assisted carrier injection, which degrades SS to 102.8 mV/dec. Under donor trap conditions, the positively charged traps attract electrons and facilitate subthreshold conduction in the n-type device, increasing SS to 95.1 mV/dec, whereas they repel holes in the pMOS, strengthening channel depletion and improving SS to 73.6 mV/dec. Consequently, it can be inferred that the traps that electrostatically repel the majority carriers enhance channel depletion in the off-state, suppress leakage current, and improve the I_ON_/I_OFF_ ratio and subthreshold characteristics, whereas traps that attract carriers weaken the channel barrier and increase subthreshold conduction.

Furthermore, the observed trends suggest the presence of a critical trap density (~ 10^12^ cm⁻²) beyond which the trap-induced electrostatic field becomes comparable to the gate-induced field, leading to significant perturbation in channel electrostatics. Hence, interface engineering and passivation of Si/dielectric interfaces are crucial, since trap polarity and density can drastically alter switching characteristics. Controlling interface traps below 10^12^ cm^-2^ would help maintain optimal digital performance.

### Impact of analog performance at Si/dielectric interface

**Fig. 8:**
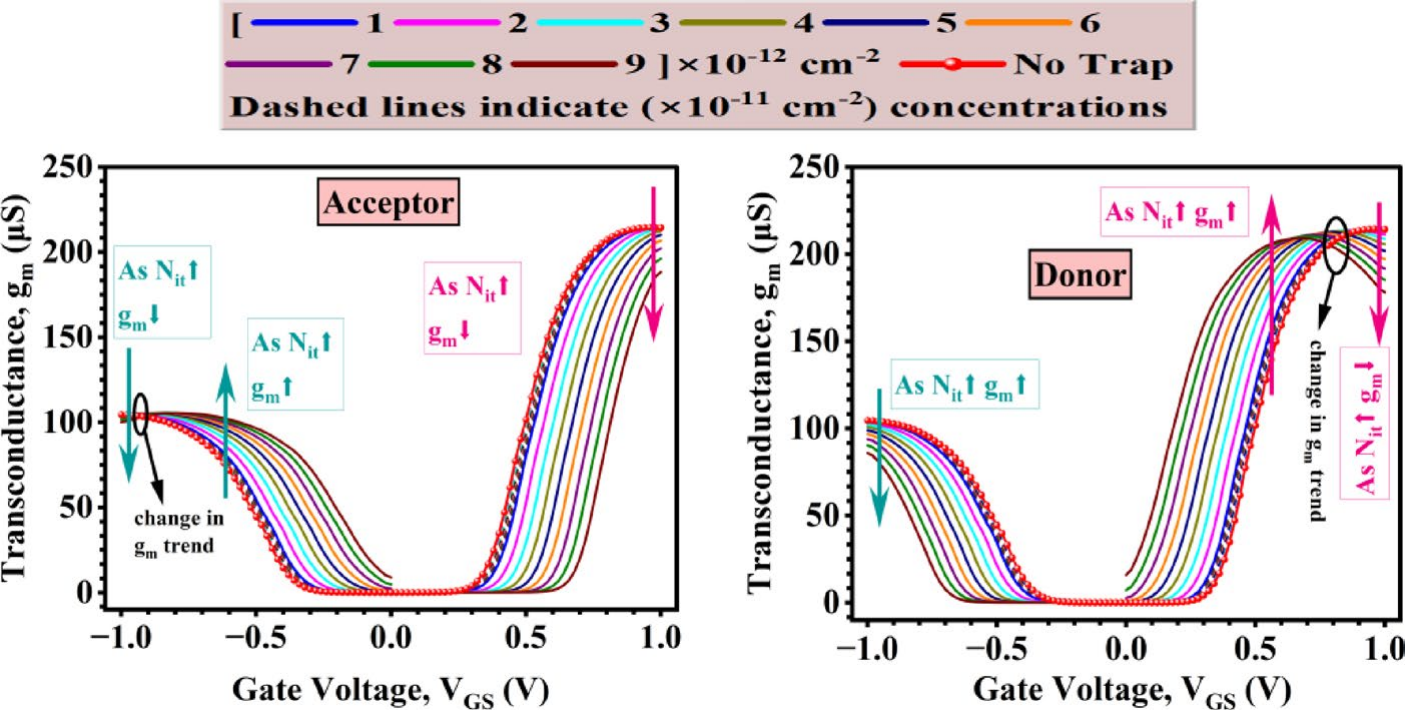
Influence of acceptor and donor N_it_ on transconductance (g_m_).

The variation in transconductance (g_m_) with N_it_ concentration is depicted in Fig. 8. Since g_m_ is governed by the sensitivity of drain current to gate-induced inversion charge, any modification in carrier density or mobility at the Si/oxide interface directly influences the g_m_ behavior. For the n-type device under acceptor trap conditions, the traps capture electrons and become negatively charged, creating a repulsive electrostatic field that weakens the electron inversion layer and reduces carrier mobility due to coulomb scattering. As a result, the gate modulation efficiency decreases and g_m_ gradually degrades with increasing trap density. The reduction remains negligible up to 3 × 10^− 12^ cm^− 2^ because the trapped charge is still small compared to the inversion charge. However, beyond 4 × 10^− 12^ cm^− 2^, the accumulated interface charge significantly perturbs the channel potential, resulting in a noticeable reduction in g_m_ (~ 11.35%). In contrast, the p-type device with acceptor traps experiences electrostatic attraction between the negatively charged traps and hole carriers, which increases hole accumulation near the interface and enhances the gate control over channel charge at moderate gate biases, leading to improved g_m_ up to V_GS_ = 0.9 V. At higher gate voltages, the increased carrier accumulation intensifies Coulomb scattering and mobility degradation, causing the observed reversal in g_m_. At high trap densities (≥ 7 × 10^− 12^ cm^− 2^), the strong negative interface charge induces partial hole accumulation even near V_GS_ =0 V, producing a finite g_m_ due to trap-assisted modulation of channel charge.

Under donor trap conditions, where traps become positively charged, a complementary behavior is observed. In the n-type device, the positive interface charge moderate gate biases (~ 0.6–0.8 V), temporarily improving attracts electrons and strengthens the inversion layer at g_m_. However, with further increase in gate bias, enhanced carrier accumulation near the interface increases coulomb scattering and mobility degradation, resulting in reduced gm at higher VGS. High donor trap densities similarly introduce residual electron concentration near the interface at low gate bias, producing a finite gm around V_GS_=0 V. Conversely, for the p-type device, the positive interface charge repels holes from the interface, weakening the inversion layer and reducing the effectiveness of gate-induced carrier modulation, which leads to a progressive decrease in g_m_ with increasing trap concentration. In all cases, the device without Si/dielectric interface traps exhibits the highest g_m_ due to the absence of trap-induced scattering and electrostatic perturbation of the channel.

**Fig. 9:**
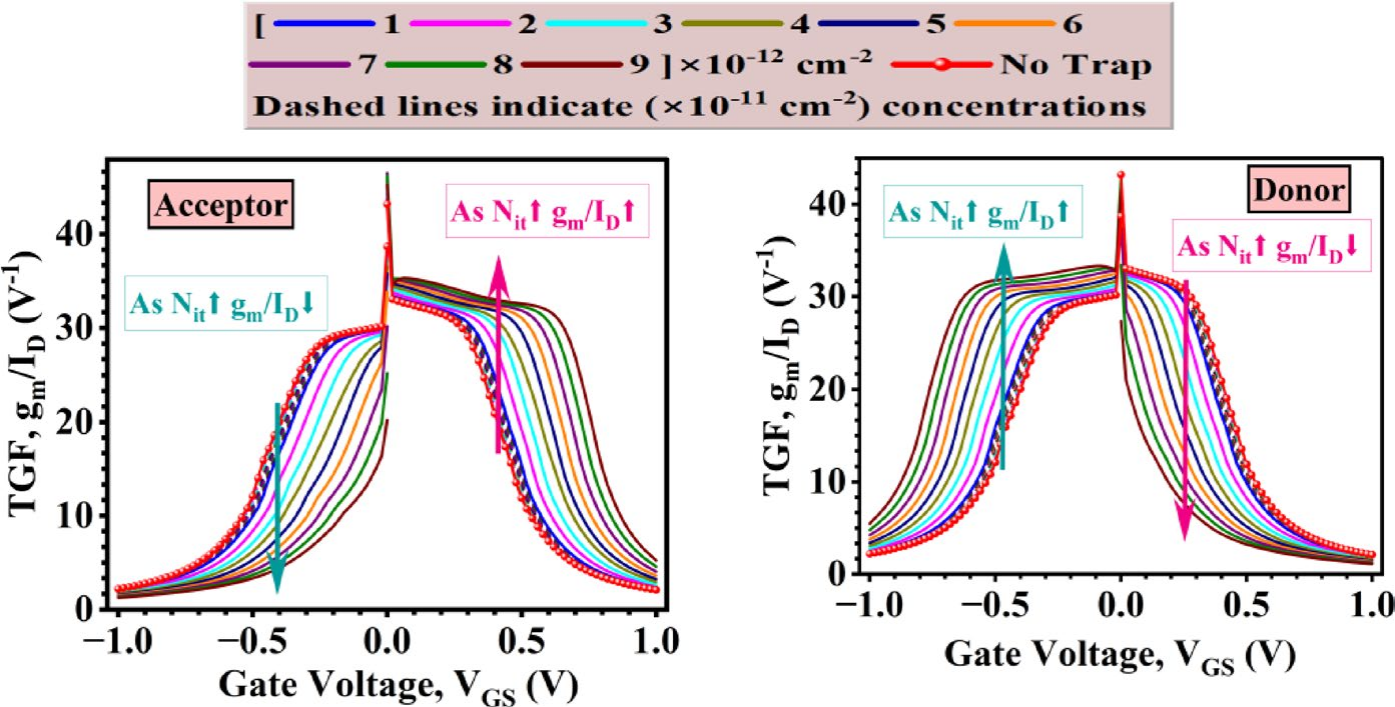
Influence of acceptor and donor N_it_ on transconductance generation factor (TGF) (g_m_/I_d_).

The transconductance generation factor, TGF (g_m_/I_D_) ratio as shown in Fig. 9 is the measure of the transconductance efficiency and is predominantly affected by the relative change between the ability to carry charges and the drain current modulated by the effect of interface trap. Under the condition of increase in acceptor trap N_it_, in the case of n-type despite decrease in the g_m_, the reduction in I_D_ will have more influence, leading to the increase in the ratio of g_m_/I_D_. In the p-type transistor, since the I_D_ enhancement is more dominant than g_m_ variation, the overall g_m_/I_D_ reduces with increasing trap density. In case of increase in donor trap N_it_, an opposite behavior is noticed. For the n-type, since the I_D_ enhancement becomes dominant compared to that of g_m_, a degradation in g_m_/I_D_ is observed. For the p-type case, as the I_D_ reduction is more pronounced than that in g_m_ decrease, the g_m_/I_D_ increases.

**Fig. 10:**
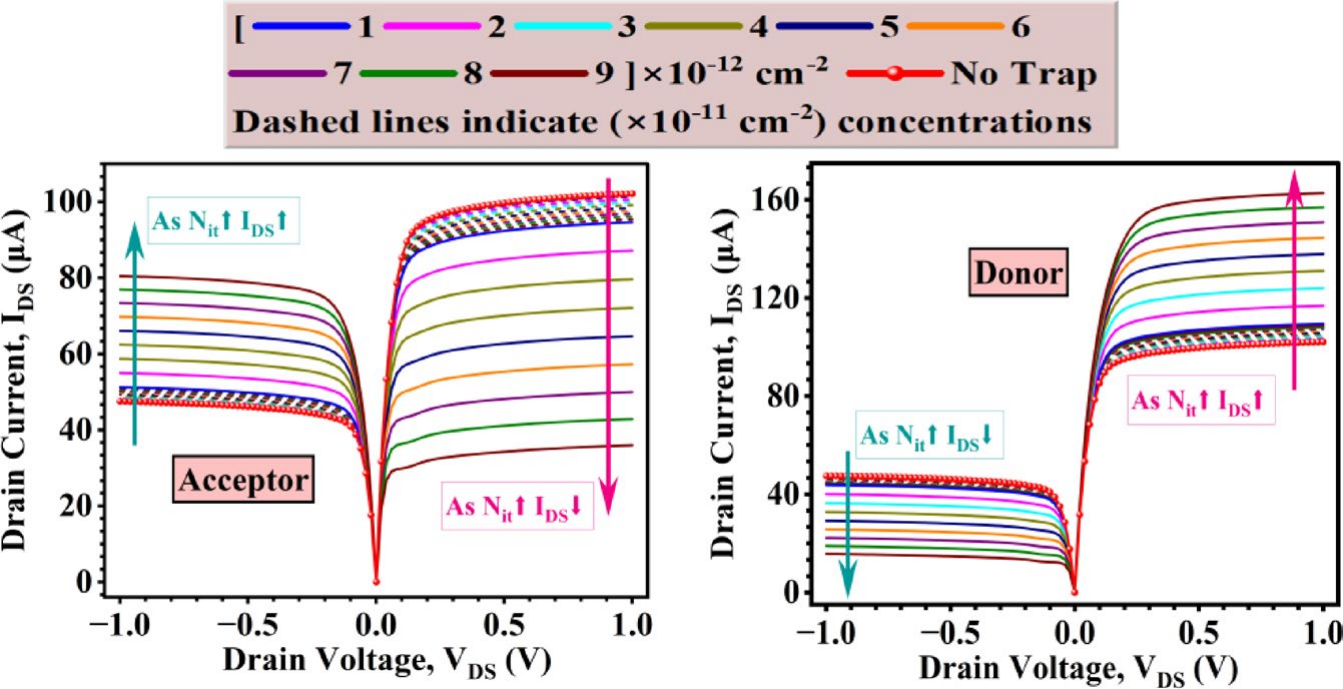
Influence of acceptor and donor N_it_ on output current characteristics (I_D_-V_DS_).

The variation in the I_D_-V_DS_ with different N_it_ is plotted in Fig. 10. For the n-type device under acceptor trap conditions, the traps capture electrons and become negatively charged, generating a repulsive electrostatic potential near the Si/oxide interface. This repulsion weakens the electron inversion layer and reduces the effective channel charge density. Additionally, the negatively charged traps introduce Coulomb scattering that degrades carrier mobility, thereby reducing the electron drift velocity under drain bias. Consequently, the drain current decreases as the acceptor trap concentration increases. In contrast, for the p-type device, the negatively charged acceptor traps electrostatically attract hole carriers toward the interface, strengthening the hole inversion layer and increasing the available channel charge. This enhanced hole accumulation improves channel conductivity, resulting in an increase in I_D_ with increasing acceptor trap density. A complementary behavior is observed under donor trap conditions, where traps capture holes and become positively charged. In the n-type device, the positive interface charge attracts electrons toward the channel interface and strengthens the electron inversion layer, thereby increasing the channel charge density and enhancing carrier transport along the channel. This leads to an increase in I_D_ as the donor trap concentration increases. Conversely, for the p-type device, the positively charged traps repel hole carriers away from the interface, weakening the hole inversion layer and reducing the effective channel charge density. The reduced hole concentration and increased scattering lower the channel conductivity, resulting in a decrease in the drain current with increasing donor trap density. These results indicate that the output current characteristics are strongly dictated by the polarity-dependent electrostatic coupling between interface trap charges and channel carriers, which modulates the effective channel conduction in the Tree-CFET structure.

**Fig. 11:**
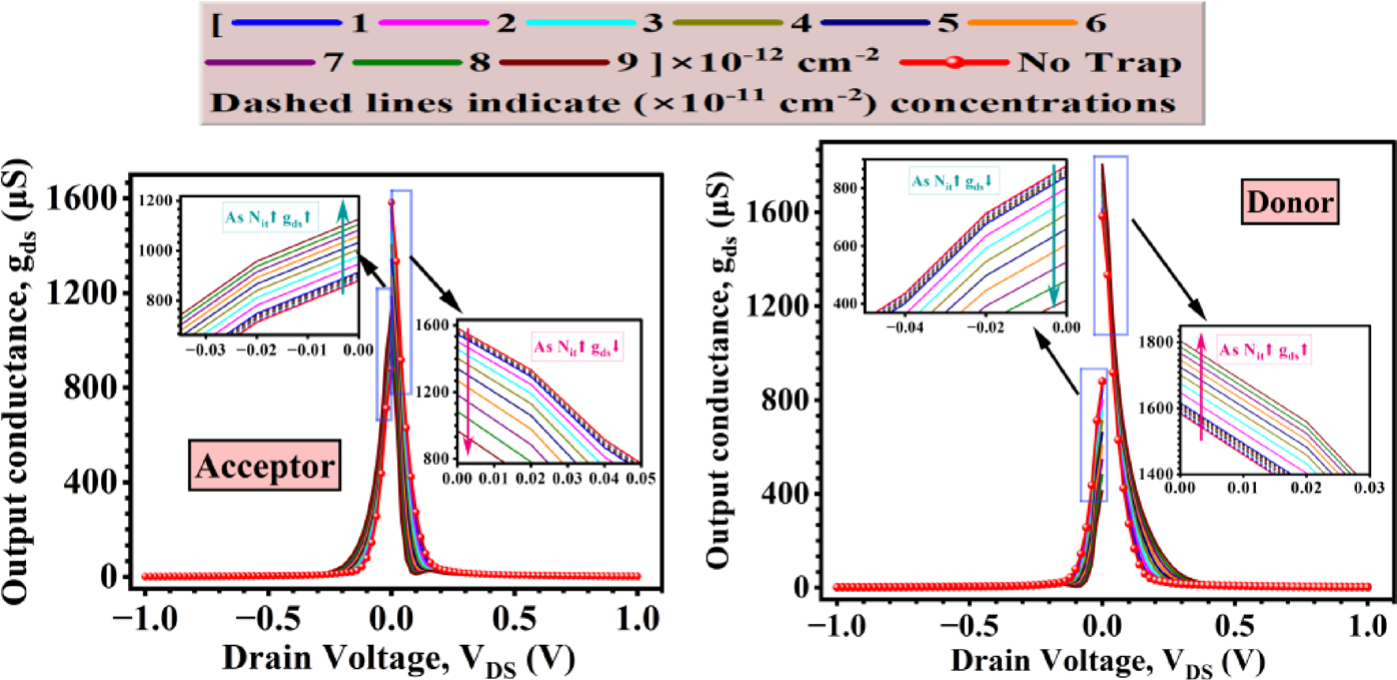
Influence of acceptor and donor N_it_ on drain to source conductance (g_ds_).

The variation in drain-to-source conductance (g_ds_) with interface trap concentration is governed by the trap-induced modulation of channel charge density and drain-field control over the channel potential as shown in Fig. 11. Since g_ds_ reflects the sensitivity of drain current to V_DS_, it is strongly influenced by the effective channel conductivity and the degree of drain-induced channel modulation. For the n-type device under acceptor trap conditions, the traps capture electrons and become negatively charged, creating a repulsive electrostatic field that weakens the electron inversion layer and reduces the effective channel carrier density. The resulting depletion of electrons near the interface reduces the drain-field penetration into the channel and weakens channel conductivity, thereby lowering the sensitivity of I_D_ to V_DS_. Consequently, g_ds_ decreases with increasing acceptor trap density.

The reduction remains marginal up to 1 × 10^− 12^ cm^− 2^ because the trapped charge is relatively small compared to the inversion charge. However, beyond this concentration, the accumulated negative interface charge significantly perturbs the channel potential and enhances carrier depletion, leading to a pronounced reduction in gds. In contrast, for the p-type device, the negatively charged acceptor traps electrostatically attract holes toward the interface, increasing the hole inversion charge and strengthening the channel conduction path. The enhanced hole concentration increases the sensitivity of the drain current to V_DS_, resulting in an increase in g_ds_, with the effect becoming significant once the trap density exceeds 1 × 10^− 12^ cm^− 2^. Under donor trap conditions, the traps capture holes and become positively charged, producing the opposite electrostatic effect. In the n-type device, the positive interface charge attracts electrons toward the channel interface, increasing the electron inversion charge and enhancing channel conductivity. This stronger conduction path allows the drain potential to more effectively modulate the channel, leading to an increase in g_ds_. Similar to the acceptor trap case, the increase remains limited at lower trap densities but becomes significant beyond 1 × 10^− 12^ cm^− 2^, where the accumulated positive interface charge strongly perturbs the channel electrostatics. Conversely, in the p-type device, the positively charged traps repel holes from the interface, weakening the inversion layer and reducing the effective channel charge density. This reduction in hole concentration diminishes the influence of the drain electric field on the channel potential, thereby decreasing the sensitivity of I_D_ to V_DS_ and resulting in a reduction in g_ds_, particularly at higher Si/dielectric interface trap densities.

**Fig. 12:**
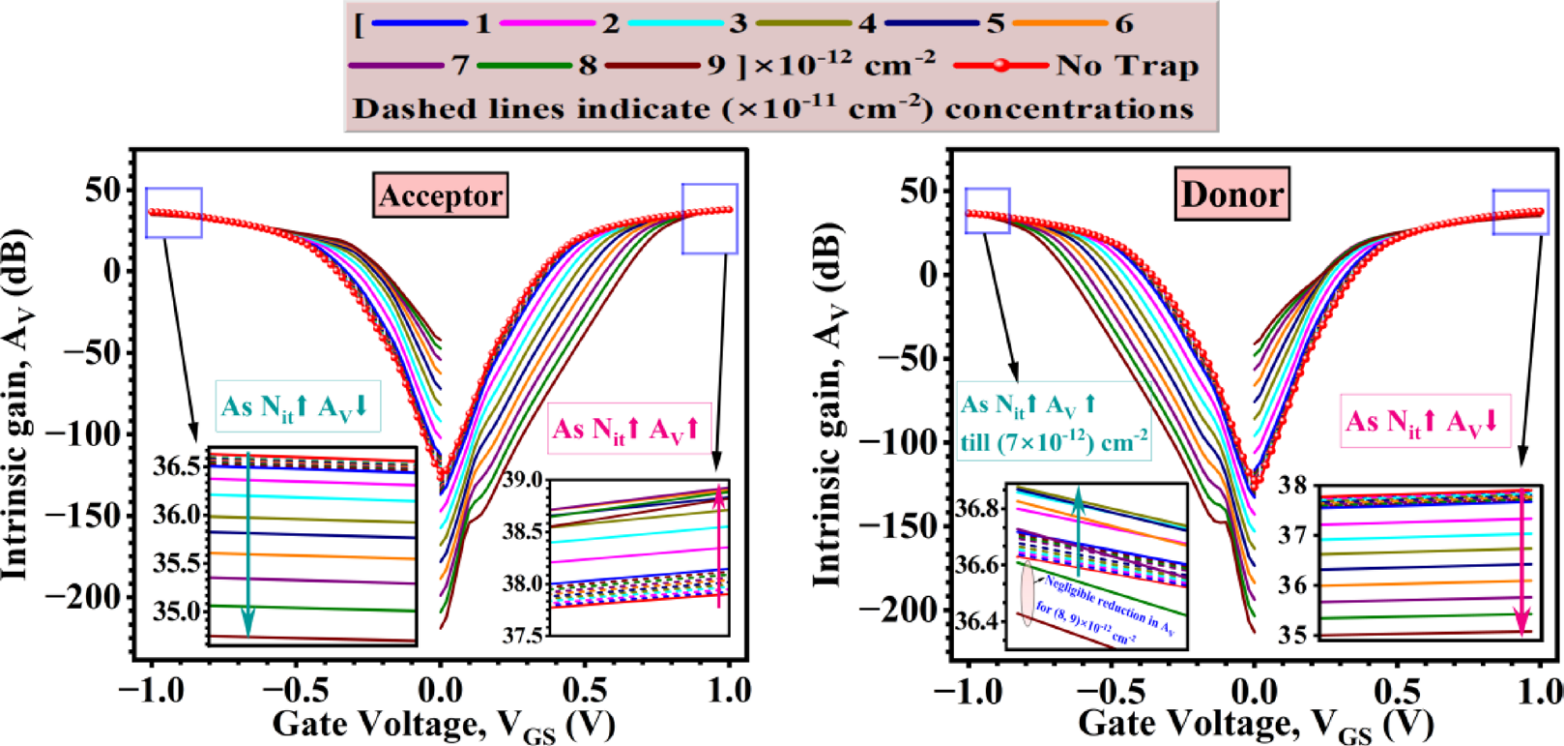
Influence of acceptor and donor N_it_ on intrinsic gain (A_V_).

The variation in intrinsic gain $\:\left({A}_{V}=20\:{log}_{10}\frac{{g}_{m}}{{g}_{ds}}\right)$ with different N_it_ is plotted in Fig. 12. For the n-type device under acceptor trap conditions, since the reduction in gds is significantly larger than that of g_m_, the ratio g_m_/g_ds_ increases marginally, resulting in a negligible improvement in AV (~ 37.9 dB to ~ 38.8 dB).

In contrast, for the p-type device, as g_ds_ incremental dominance is higher moderately affecting g_m_, the g_m_/g_ds_ ratio reduces, leading to a small degradation in A_V_ (~ 36.6 dB to ~ 34.7 dB). Under donor trap conditions, in the n-type device, A_V_ reduces from ~ 37.8 dB to ~ 35 dB due to the combined effect of reduction in g_m_ and increase in g_ds_. Conversely, in the p-type device, the g_ds_ reduces more prominently than g_m_ leading to a negligible improvement in A_V_ (~ 36.6 dB to ~ 36.9 dB). Overall, the relatively small variation in intrinsic gain indicates that the trap-induced changes in g_m_ and g_ds_ partially compensate each other, maintaining nearly stable analog gain characteristics in the device.

### Impact of RF performance at Si/dielectric interface

**Fig. 13:**
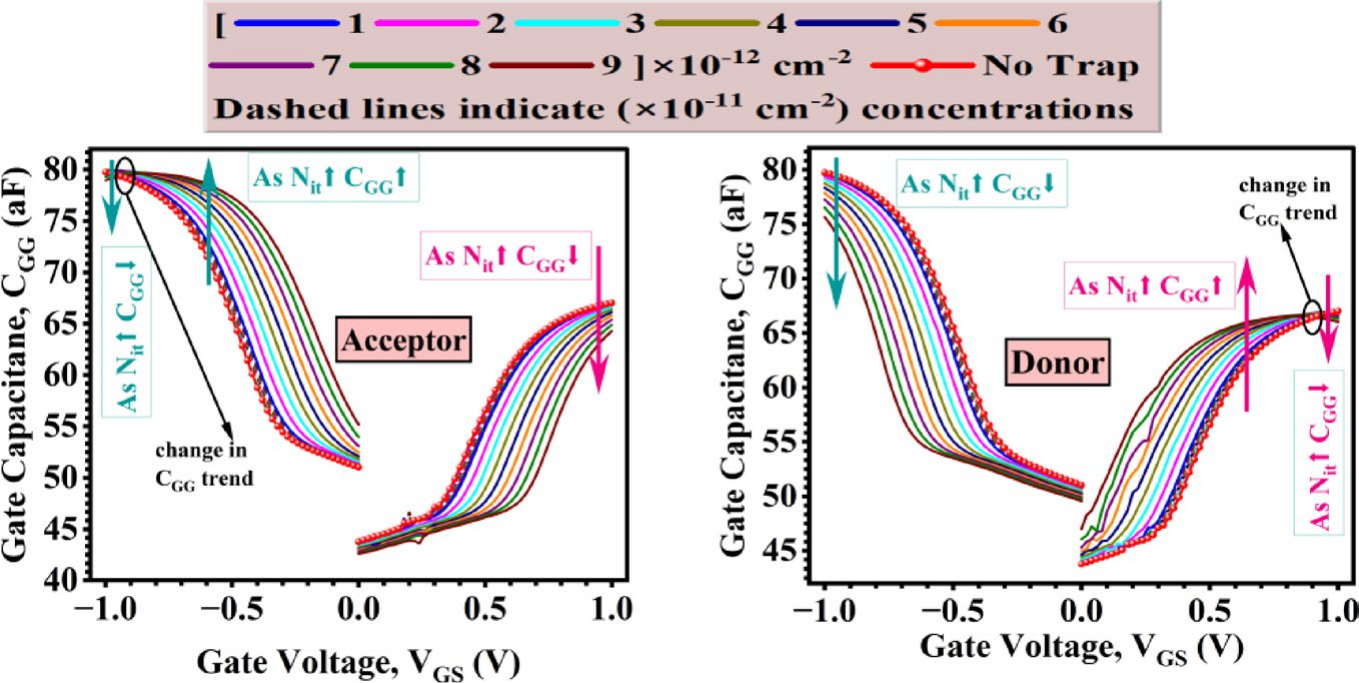
Influence of acceptor and donor N_it_ on gate capacitance (C_GG_).

The variation in gate capacitance (C_GG_) with N_it_ concentration is depicted in Fig. 13. For the n-type device under acceptor trap conditions, the traps capture electrons and become negatively charged, producing a repulsive electrostatic field that reduces the electron concentration near the interface. This reduction in inversion charge weakens the gate-to-channel coupling and slightly decreases C_GG_ (67 aF to 64 aF) as the trap concentration increases from 1 × 10^− 11^ cm^− 2^ to 9 × 10^− 12^ cm^− 2^. The reduction is relatively small because the dominant oxide capacitance still governs the total gate capacitance, while the trap-induced electrostatic perturbation only marginally modifies the inversion charge distribution. In contrast, for the p-type device, the negatively charged acceptor traps attract holes toward the interface, enhancing the hole accumulation and increasing the differential channel charge with respect to gate voltage. Consequently, C_GG_ increases with trap concentration up to moderate gate bias (V_GS_=0.9 V), where the inversion charge becomes dominant and all curves converge. Beyond this bias, strong hole accumulation near the interface increases coulomb scattering and mobility degradation, which weakens the incremental modulation of channel charge with gate voltage, resulting in a slight reduction in C_GG_ from 79.7 aF to 78.6 aF at higher trap densities. Under donor trap conditions, where traps become positively charged, the electrostatic interaction reverses. In the n-type device, the positive interface charge attracts electrons toward the interface, strengthening the electron inversion layer and increasing the sensitivity of channel charge to gate bias. This leads to an increase in C_GG_ with trap concentration up to V_GS_=0.9 V. At higher gate voltages, however, the increased electron accumulation near the interface enhances coulomb scattering and reduces the incremental charge modulation with gate bias, causing a reversal in the trend and a gradual reduction in C_GG_ from 79.7 aF to 75.6 aF.

Conversely, for the p-type device, the positively charged donor traps repel holes from the interface and slightly weaken the hole inversion layer, leading to a small reduction in the differential channel charge and consequently a marginal decrease in CGG from 67 aF to 66 aF. Overall, the relatively small variation in C_GG_ indicates that the dominant oxide capacitance largely governs the gate capacitance, while trap-induced electrostatic effects introduce only minor perturbations to the channel charge distribution.

**Fig. 14:**
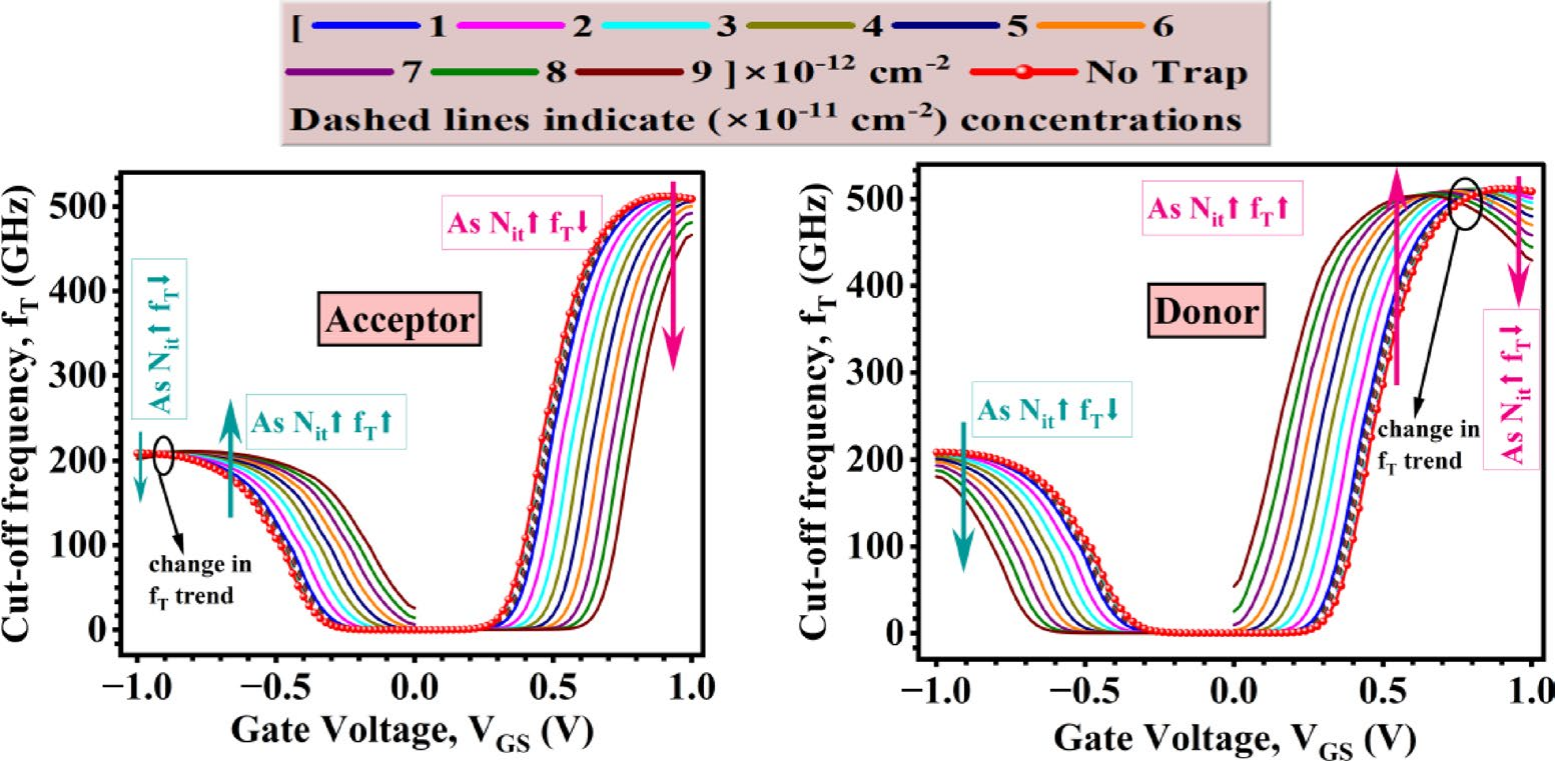
Influence of acceptor and donor Nit on cutoff-frequency (f_T_).

The variation in the cutoff-frequency $\:\left({f}_{T}=\frac{{g}_{m}}{2\pi\:{C}_{GG}}\right)$ with different N_it_ is plotted in Fig. 14. The f_t_ represents the intrinsic speed of the device and is governed by the ratio between the transconductance and the total gate capacitance, which correspond to the carrier transport capability and the charge storage in the channel, respectively.

In the present device, the interface traps primarily influence carrier transport through electrostatic perturbation and coulomb scattering, which directly modifies the inversion charge and carrier mobility, thereby strongly affecting g_m_. In contrast, the variation in C_gg_ with trap concentration is relatively small because the oxide capacitance remains the dominant component of the total gate capacitance, and the trap-induced perturbation only slightly alters the channel charge distribution. As a result, the denominator of the f_t_ expression remains nearly constant, while the numerator g_m_ experiences more noticeable variations due to trap-induced modulation of carrier mobility and inversion charge density. Consequently, f_T_ predominantly follows the same trend as g_m_, indicating that the RF speed of the device is primarily limited by carrier transport characteristics rather than gate capacitance variations under Si/dielectric interface trap perturbation.

**Fig. 15:**
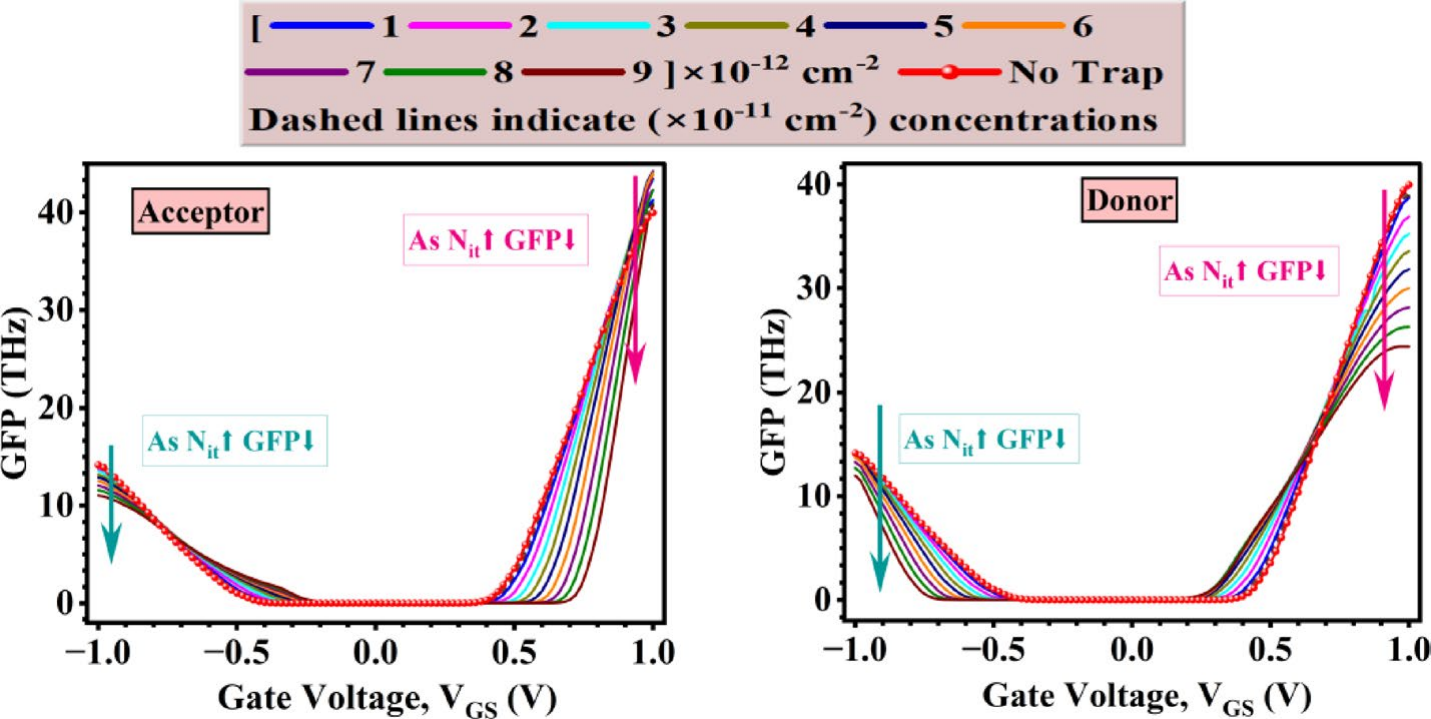
Influence of acceptor and donor N_it_ on gain frequency product (GFP).

The Gain Frequency Product $\:\left(GFP=\:\frac{{g}_{m}}{{g}_{ds}}\times\:{f}_{T}\right)$ as shown in Fig. 15 is an important performance measure that incorporates the intrinsic gain and high frequency capabilities of devices into one metric and makes it very useful in comparing transistor technologies for analog and RF design. This measure clearly brings out the tradeoff between the gain and bandwidth. Higher values of GFP reflect a better performing transistor technology because they allow amplifier circuits to provide more gain at higher frequencies, thus providing a more useful function in low noise amplifiers and operational amplifiers. In both cases of increasing acceptor and donor N_it_, the GFP declines for both n-type and p-type due to the resulting decrease in both f_T_ and g_m_ when compared to the g_ds_.

### Evaluation of CMOS inverter circuit performance

**Fig. 16:**
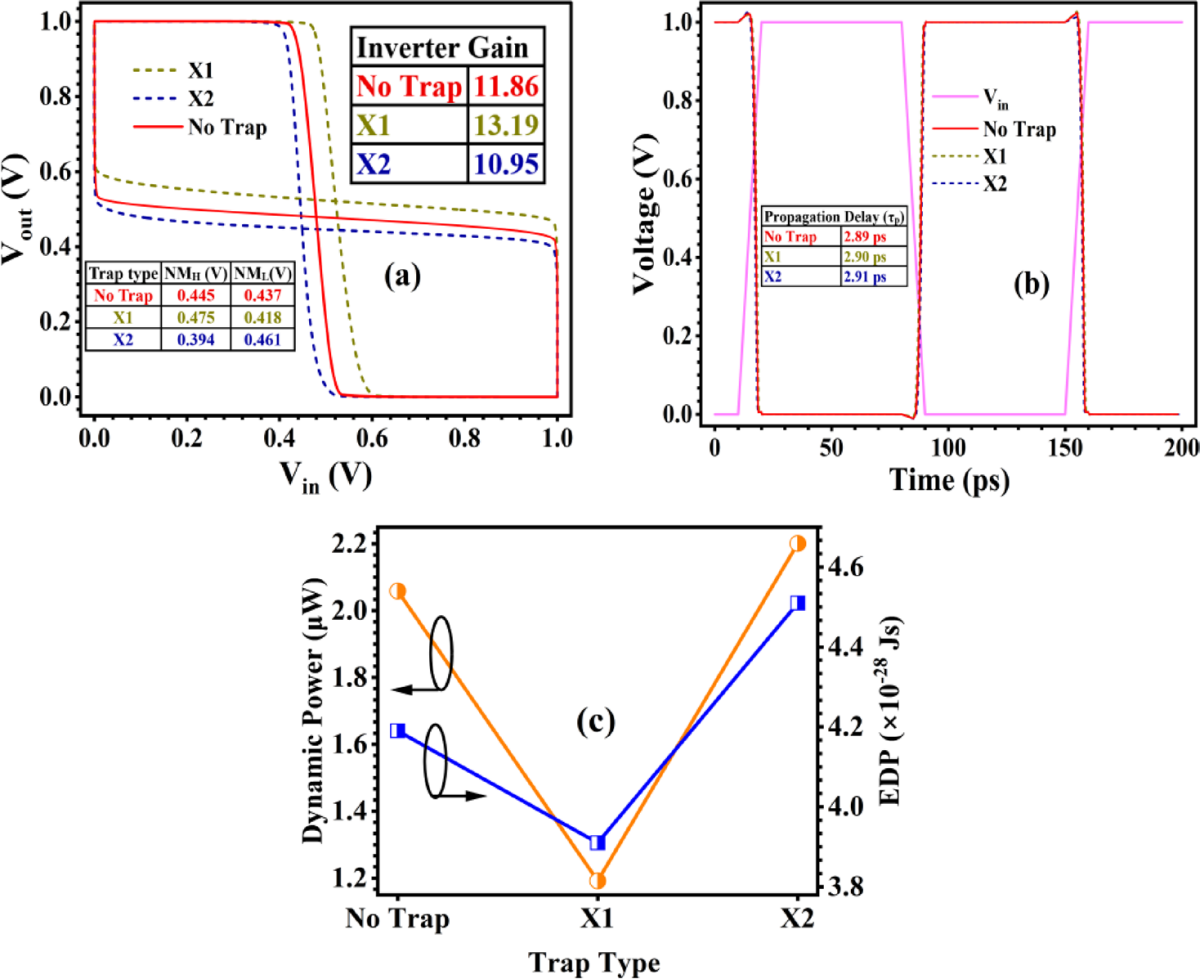
CMOS inverter characteristics (**a**) Butterfly curves (**b**) Transient analysis to verify CMOS inverter operation (**c**) Dynamic power and EDP.

The effect of voltage transfer characteristics (VTC) and transient analysis of the CMOS inverter is shown in Fig. 16a,b respectively. Here X1 and X2 configurations indicates the incorporation of acceptor and donor traps respectively in the CFET inverter. The noise margin, propagation delay (τ_p_), and voltage gain are extracted to examine the impact of trap-induced electrostatic perturbations on inverter switching characteristics. For the no-trap case, the inverter exhibits a noise margin with NM_H_=0.445 V and NM_L_=0.437 V, indicating nearly symmetric switching robustness for both logic states. The τ_p_ is observed to be 2.89 ps, reflecting efficient charge transfer between the complementary devices due to balanced carrier transport. The corresponding voltage gain of 11.86 demonstrates strong VTC and sharp switching behavior, confirming that the trap-free Tree-CFET provides stable inverter operation with well-balanced pull-up and pull-down strengths. With X1, the noise margin shifts to NM_H_=0.475 V and NM_L_=0.418 V. The increase in NM_H_ combined with the slight reduction in NM_L_ indicates a modest asymmetry in the switching threshold. This behavior arises because acceptor traps introduce negative interface charge, which electrostatically repels electrons in the n-type device while attracting holes in the p-type device. Consequently, the p-device conduction becomes relatively stronger, shifting the switching point and slightly improving the inverter voltage gain to 13.19. The τ_p_ shows only a marginal increase to 2.90 ps, indicating that the trap-induced electrostatic perturbation does not significantly degrade the transient switching speed at this trap concentration. In contrast, the presence of donor traps results in NM_H_=0.394 V and NM_L_=0.461 V, reflecting the opposite shift in switching balance. Donor traps introduce positive interface charge, which strengthens the electron conduction in the n-type device while weakening the hole conduction in the p-type device. As a result, the pull-down network becomes relatively stronger, shifting the inverter switching point and reducing the overall voltage gain to 10.95. The τ_p_ slightly increases to 2.91 ps, which can be attributed to trap-induced scattering and minor imbalance between the complementary devices. Consequently, the results indicate that interface trap polarity primarily influences the switching symmetry and gain of the inverter by altering the relative strengths of the pull-up and pull-down networks, while the τ_p_ remains nearly unchanged due to the strong electrostatic control of the Tree-CFET architecture.

Additionally, the dynamic power and energy delay (EDP) product of the CMOS inverter is plotted in Fig. 16c. The dynamic power is the power consumed during the switching of the CMOS inverter, which means when the output changes its state from ‘0’ to ‘1’ or ‘1’ to ‘0’. The dynamic power happens due to the charging and discharging of the load capacitance which is given by:

$$\:Dynamic\:power=\:{C}_{L}.{V}_{DD}^{2}.f$$

where $\:{C}_{L}$ = load capacitance, $\:{V}_{DD}$= supply voltage, * f* = switching frequency.

The Energy Delay Product $\:\left(EDP=\frac{{C}_{L}.{V}_{DD}^{2}.\tau\:}{2}\right)$ combines both the power efficiency and speed into one single measure and evaluates the relationship between power utilization and speed. Ideally, both the EDP and dynamic power should be lower for better CMOS inverter performance. It can be noticed that both the EDP and dynamic power are found to be lower for X1 when compared to No trap and X2 case. The decrease in dynamic power for X1 is mainly due to the fact that the drive current and switching effects become weak owing to carrier depletion induced by traps in the channel. The charging/discharging current through the capacitive load, which contributes to dynamic power, becomes weak because of the above factors by ~ 42%. However, for the case of donor trap, the power consumption increases by ~ 6.8%. For X1, the effect of reduced power dissipation outweighs the effect of increased delay variation, which causes a decrease in EDP. For X2, there is an increase in switching power because of greater leakage current, which increases EDP. From these observations, it can be inferred that acceptor traps have better energy-efficient switching, while donor traps affect the proposed design’s power-delay trade-off negatively.

## Conclusion

The presented analysis demonstrates that the performance of Tree-shaped CFETs is strongly governed by the polarity and density of Si/dielectric interface traps, which modulate channel electrostatics and carrier transport through trap-carrier interactions. Traps that electrostatically repel the dominant carriers effectively enhance channel depletion in the off-state, suppress trap-assisted leakage, and significantly improve the switching ratio, whereas traps that attract carriers facilitate subthreshold conduction and degrade switching behavior due to enhanced leakage currents. The study further indicates that the switching characteristics remain robust as long as the Si/dielectric interface trap density is maintained within a moderate range. Once the trap density exceeds approximately 4 × 10^12^ cm^− 2^, trap-assisted carrier generation and electrostatic perturbation of the channel become pronounced, leading to substantial degradation in switching performance. In contrast, the analog and RF characteristics exhibit comparatively lower sensitivity to interface traps because the trap-induced variations in transconductance and output conductance partially compensate each other, and the gate capacitance remains largely unchanged, preserving intrinsic gain and cut-off frequency. Also, the CMOS inverter demonstrated stable operation with noise margins of ~ 0.418–0.475 V, propagation delay around ~ 2.9 ps, and inverter gain up to ~ 13.19 under trap conditions. Consequently, these findings highlight the importance of Si/dielectric interface engineering and trap density control in CFETs to enable scalable, high-performance, and reliable operation for future sub-3 nm CMOS technology nodes. Also, it is important to note that the present study is based on calibrated TCAD simulations and does not account for process-induced variability and long-term reliability effects such as BTI and HCI. Therefore, the quantitative trends should be interpreted within these modeling assumptions.

## Supplementary Information

Below is the link to the electronic supplementary material.

Supplementary Material 1

## Data Availability

The authors confirm that all data are included within the manuscript.
