# Supplementary material for: Role of Si/dielectric interface traps on the reliability of tree-shaped complementary FETs for sub-3 nm technology nodes
Source: Sci Rep. 2026 Jun 16;16:25503. doi: 10.1038/s41598-026-57907-2 (PMC13476250; doi:10.1038/s41598-026-57907-2)
Supplement: Supplementary file 1 [file 41598_2026_57907_MOESM1_ESM.docx]

| **ACCEPTOR**   | **DONOR**   |
| --- | --- |
|  |  |
|  |  |

**Figure S1.** Electron and hole current densities distribution with N_it_ ranging from 1×10^-12^ cm^-2^ to 9×10^-12^ cm^-2^ to validate the ON current behaviour in acceptor and donor traps.

**Table S1.** I_ON_/I_OFF_ ratio of Acceptor traps for Tree-shaped CFETs

| **Trap conc.** | **I_ON_/I_OFF_ nMOS** | **I_ON_/I_OFF_ pMOS** |
| --- | --- | --- |
| 1 × 10^12^ | 1.87 × 10^7^ | 8.80 × 10^5^ |
| 2 × 10^12^ | 5.90 × 10^7^ | 2.79 × 10^5^ |
| 3 × 10^12^ | 1.85 × 10^8^ | 8.86 × 10^4^ |
| 4 × 10^12^ | 5.49 × 10^8^ | 2.82 × 10^4^ |
| 5 × 10^12^ | 1.82 × 10^9^ | 9.09 × 10^3^ |
| 6 × 10^12^ | 5.67 × 10^9^ | 2.99 × 10^3^ |
| 7 × 10^12^ | 1.74 × 10^10^ | 1.04 × 10^3^ |
| 8 × 10^12^ | 5.23 × 10^10^ | 4.06 × 10^2^ |
| 9 × 10^12^ | 1.50 × 10^11^ | 1.83 × 10^2^ |

**Table S2.** I_ON_/I_OFF_ ratio of Donor traps for Tree-shaped CFETs

| **Trap conc.** | **I_ON_/I_OFF_ nMOS** | **I_ON_/I_OFF_ pMOS** |
| --- | --- | --- |
| 1 × 10^12^ | 1.90 × 10^6^ | 8.76 × 10^6^ |
| 2 × 10^12^ | 6.07 × 10^5^ | 2.77 × 10^7^ |
| 3 × 10^12^ | 1.94 × 10^5^ | 8.77 × 10^7^ |
| 4 × 10^12^ | 6.19 × 10^4^ | 2.78 × 10^8^ |
| 5 × 10^12^ | 1.98 × 10^4^ | 8.77 × 10^8^ |
| 6 × 10^12^ | 6.39 × 10^3^ | 2.75 × 10^9^ |
| 7 × 10^12^ | 2.11 × 10^3^ | 8.48 × 10^9^ |
| 8 × 10^12^ | 7.28 × 10^2^ | 2.54 × 10^10^ |
| 9 × 10^12^ | 2.80 × 10^2^ | 7.15 × 10^10^ |
